# Effect of Different Amino Acids and Heating Conditions on the Formation of 2-Amino-1-methyl-6-phenylimidazo[4,5-b]pyridine (PhIP) and Its Kinetics Formation Using Chemical Model System

**DOI:** 10.3390/molecules24213828

**Published:** 2019-10-24

**Authors:** Ainaatul Asmaa Ishak, Jinap Selamat, Rabiha Sulaiman, Rashidah Sukor, Emilia Abdulmalek, Nuzul Noorahya Jambari

**Affiliations:** 1 Department of Food Science, Faculty of Food Science and Technology, Universiti Putra Malaysia, Serdang 43400, Selangor, Malaysia; aina17ain@gmail.com (A.A.I.); syidahsukor@gmail.com (R.S.); noorahya@upm.edu.my (N.N.J.); 2 Laboratory of Food Safety and Food Integrity, Institute of Tropical Agriculture and Food Security, University Putra Malaysia, Serdang 43400, Selangor, Malaysia; 3 Department of Food Technology, Faculty of Food Science and Technology, Universiti Putra Malaysia, Serdang 43400, Selangor, Malaysia; rabiha@upm.edu.my; 4 Department of Chemistry, Faculty of Science, Universiti Putra Malaysia, Serdang 43400, Selangor, Malaysia; emilia@upm.edu.my

**Keywords:** kinetic study, chemical model system, amino acids and 2-amino-1-methyl-6-phenylimidazo[4,5-b]pyridine (PhIP)

## Abstract

The formation of 2-amino-1-methyl-6-phenylimidazo[4,5-b]pyridine (PhIP) was investigated using a kinetic study approach as described by first-order, Arrhenius, and Eyring equations. Chemical model systems with different amino acid precursors (proline, phenylalanine, and glycine) were examined at different times (4, 8, 12, and 16 min) and temperatures (150, 180, 210, 240, and 270 °C). PhIP was detected using high-performance liquid chromatography equipped with fluorescence detector (HPLC-FLD). The good fit in first-order suggested that PhIP formation was influenced by the types of amino acids and PhIP concentration significantly increased with time and temperature (up to 240 °C). PhIP was detected in proline and phenylalanine model systems but not in the glycine model system. The phenylalanine model system demonstrated low activation energy (Ea) of 95.36 kJ/mol that resulted in a high rate of PhIP formation (great amount of PhIP formed). Based on the ∆S^‡^ values both proline and phenylalanine demonstrated bimolecular rate-limiting steps for PhIP formation. Altogether these kinetic results could provide valuable information in predicting the PhIP formation pathway.

## 1. Introduction

Heterocyclic amines (HCAs) are known as food mutagens/carcinogens that are formed when heat is introduced to muscle food such as meat, poultry, and fish during food preparation [1,2,3]. The HCAs are formed in cooked food when the cooking temperature reaches greater than 150 °C [2,4]. HCAs can be divided into two classes based on their formation and chemical conformation, namely the aminoimidazo-azaarenes (IQ-types) and the amino-carboline (non-IQ-types) [4,5]. The aminoimidazo-azaarenes, commonly known as AIA-type, are the major subclass of HCAs found in human diet [6]. The AIA-type HCAs include:

- 2-amino-3-methylimidazo[4,5-f]quinoline (IQ)
- 2-amino-3,4-dimethyl-3H-imidazo[4,5-f]quinolone (MeIQ)
- 2-amino-3,8-dimethylimidazo[4,5-f]quinoxaline (MeIQx)
- 2-amino-3,7,8-trimethyl-3H-imidazo[4,5-f]quinoxaline (DiMeIQx)
- 2-amino-1-methyl-6-phenylimidazo[4,5-b]pyridine (PhIP)

All AIA-type HCAs have an imidazole ring in their structures that originates from creatine/creatinine. The imidazole ring, however, is not found in amino-carboline as its formation involves pyrolysis of amino acids at temperature above 300 °C [5]. PhIP, in particular, is abundant among the AIA compounds in cooked food [7]. According to the International Agency for Research on Cancer (IARC), PhIP is classified as probable carcinogen and may cause human cancer (Class 2B) [8] with its LD_50_ estimated to be 3 mg/kg [9]. Epidemiological studies have demonstrated that high daily intake of PhIP can cause colon, prostate, and breast cancers [10,11,12]. Evidence has shown that the ability of PhIP forming DNA-adduct by altering the DNA sequence in human body is the main cause of cancer [6,13]. Alarmingly, the estimated average daily intake of PhIP among the Malaysian population is relatively high at 121.4 ng/g per capita day^−1^ [1], which is comparable to the mean daily intake of PhIP in the USA that varies from 78.2 to 1029 ng/g per capita day^−1^, with the highest source of PhIP intake from meat [1]. Other countries such as Japan (5–300 ng/g), Sweden (72 ng/g), and Spain (344 ng/g) also exhibited a high daily intake of PhIP per capita day^−1^ [1]. These findings further indicate the high exposure of PhIP in human diet regardless of country of origin.

Several external factors have been shown to contribute towards the formation of PhIP in food. For example, meat types affect the amount of PhIP formed, as evidently demonstrated by a previous study where a total of 5.27, 9.20, and 6.06 ng/g of PhIP was detected in fried beef, pork, and chicken samples, respectively [14]. Moreover, levels of PhIP detected increased with doneness of chicken and beef satays up to 7.7 and 11.30 ng/g, respectively. The use of different type of sugars during marinating demonstrated different PhIP levels up to 6.26–7.44 ng/g using table sugar, 6.08–7.74 ng/g using brown sugar, and 9.52–10.22 ng/g using honey for grilled marinated chicken [3]. Food cooked using a pan-frying method formed a higher PhIP level than roasting [15]. Moreover, Iwasaki et al. [16] reported high PhIP content of 47.3 and 31.8 ng/g, respectively in both chicken and beef grilled directly over an open flame.

Other than meat types, the marinating process, and cooking conditions, the formation of PhIP in cooked food can also be induced by the naturally occurring precursors in the food itself such as amino acids, reducing sugars, and creatine/creatinine [17]. Chemical model and food model systems have been widely used to study the chemical and physical conditions involved in the formation of PhIP. The chemical model system that consists mainly of the major precursors such as reducing sugars, creatinine, and amino acids has been used to study the precursor effects on PhIP formation. A study done by Skog et al. reported that phenylalanine was the main amino acid responsible for PhIP formation [18] and PhIP has also been successfully extracted from mixture of heated creatinine, glucose and phenylalanine refluxed for 2 h at 128 °C in diethylene glycol with 14% water (DEG) [19]. A dry heated model system comprised of phenylalanine and leucine pyrolysis for 1 h at 200 °C was proposed by Övervick et al. [20] to study PhIP formation. Besides that, other amino acids also have been demonstrated to involve in PhIP formation [20]. Johanson et al. [21] successfully isolated PhIP from a tyrosine and leucine model system.

In addition to the model system, kinetic study was used to examine the rate of reactions and variables effect on HCA formation. Nonetheless, most of the model systems were based on a meat and food model system. A meat juice and meat model system were used by Arvidsson et al. [22] and Ahn and Grün [23], respectively, to study the kinetic formation of HCAs including PhIP. Hwang and Ngadi [24] used meat emulsion with different fat contents to investigate the kinetic formation of HCAs compounds. However, there have been limited data on the use of a chemical model system in studying the kinetic effect on PhIP or HCA formation. Moon and Shin [25] reported the kinetic formation of PhIP using a chemical model system of phenylalanine heated at 125–200 °C. Moreover, Arvidsson et al. [26] demonstrated PhIP and other HCAs formation using a mixture of amino acids model system. Each of the single precursors (amino acids, sugar, and creatinine) influences the rate formation of PhIP in the meat systems. The chemical model system can be a useful technique to study the effects of single precursor (amino acids, sugars, and creatinine) as no other food matrices are involved and could interfere with the reaction. By adding the kinetic study in the chemical model system, the rate formation of PhIP from an individual precursor can be determined and studied in more detail.

Therefore, the objective of this paper was to study the kinetic formation of PhIP by using single amino acids with different chemical structures as the precursors heated at various temperatures (150–270 °C). In this study, phenylalanine, proline, and glycine were selected as the amino acid precursors mainly due to their linear, cyclic, and aromatic molecular structures. The data on the kinetic parameter and model system could provide insights towards the role of phenylalanine, proline, and glycine with different constituents on PhIP formation.

## 2. Results and Discussion

### 2.1. PhIP Identification and Quality Parameter

**Figure 1:**
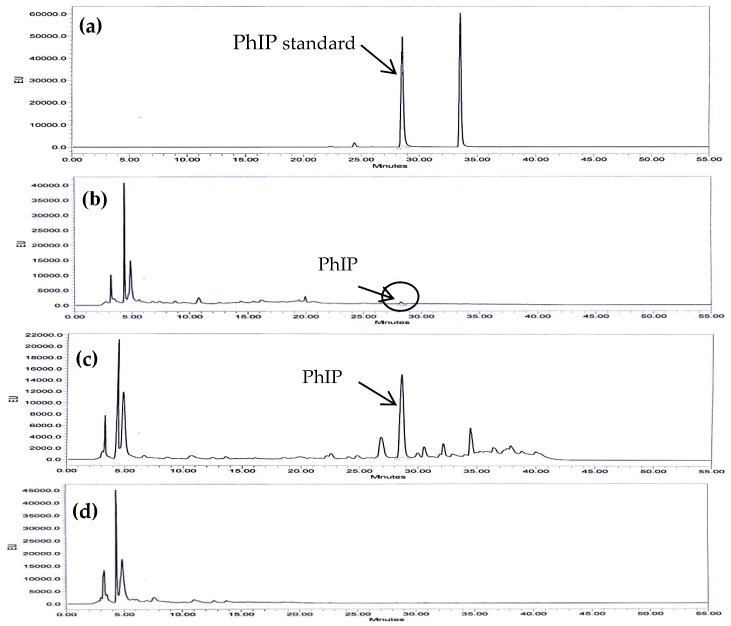
Typical high performance liquid chromatography (HPLC-FLD) chromatograms of 2-amino-1-methyl-6-phenylimidazo[4,5-b]pyridine (PhIP) at 210 °C for 16 min: (**a**) PhIP standard; (**b**) PhIP in the proline model system; (**c**) PhIP in the phenylalanine model system; (**d**) PhIP in the glycine model system.

Figure 1 compares the HPLC-FLD chromatograms of standards and the three model systems at 240 °C for 4 min. A PhIP peak was detected in proline and phenylalanine model systems at 28.24–28.52 min but no PhIP peak was detected in the glycine model system. To examine the quality of the method performance, the Limit of Detection (LOD) and Limit of Quantification (LOQ) of PhIP that were calculated based from the standard deviation (SD) of response and slope were determined to be 0.0002 and 0.002 ng/mL, respectively. A linear calibration curve for PhIP was constructed based on the peak area. The correlation coefficient (R^2^) for the calibration graph was 0.9921, indicating a strong relationship between peak area ratio and the concentration of PhIP.

### 2.2. Effects of Time and Temperature

**Figure 2:**
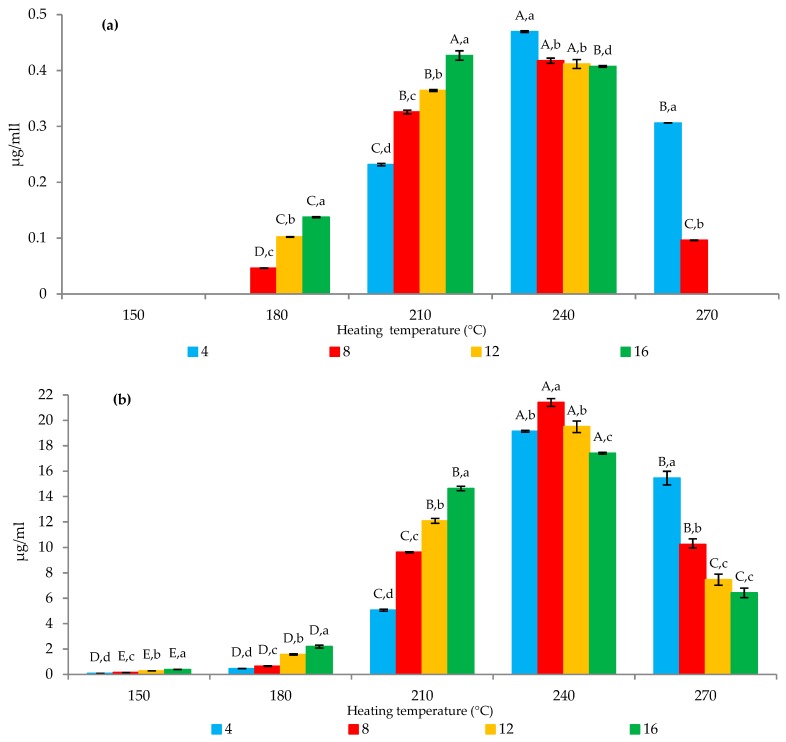
Effects of time and temperature on PhIP formation in (**a**) proline model system and (**b**) phenylalanine model system. A–E: mean between samples for each temperature that share the same letter are not significantly different (*p* > 0.05). a–d: mean between samples for each heating time that share the same letter are not significantly different (*p* > 0.05).

The heating process of the mixture containing creatinine, glucose, and single amino acids successfully formed PhIP in proline and phenylalanine model systems only (Figure 2) but not in the glycine model system. These results were consistent with a previous study [25] where PhIP was not formed in a model system containing a mixture of creatinine, glucose, and glycine. Proline and phenylalanine model systems demonstrated a significant (*p* < 0.05) interaction between temperature and time changes on PhIP formation.

At 150 °C, no formed PhIP was detected in the proline model system at any range of time. As the temperature increased to 180 °C, 0.04 µg/mL of PhIP was detected after 8 min of heating. The amount was significantly (*p* < 0.05) increased with increased time and temperature. At 210 °C, 0.23–0.43 µg/mL of PhIP was detected as the mixture was heated for 4–16 min. The highest amount of PhIP formed was 0.47 µg/mL at 240 °C after 4 min of heating. Nevertheless, as the heating continued to 8 min, the amount of PhIP reduced with increased time and temperature. There was 11% reduction observed between 4 to 8 min of heating at 240 °C. At 240 °C, no significant difference (*p* > 0.05) on the PhIP amounts detected at 8, 12, and 16 min. These findings were in agreement with a previous study by Chiu and Chen [27] where they revealed that PhIP had the lowest thermal stability among 15 HCAs compounds. Therefore, we concurred that the reduced concentration of PhIP observed in this study was due to PhIP degradation.

PhIP demonstrated faster formation in the phenylalanine model system than the proline model system where 0.15 µg/mL of PhIP started forming after 4 min of heating at 150 °C. The amount was significantly (*p* < 0.05) increased with increased time and temperature until 240 °C (8 min), which recorded 21.41 µg/mL as the highest amount. After 12 min of heating, 9% degradation of PhIP was observed, which reduced the amount of PhIP formed to 19.50 µg/mL. The PhIP amount continually reduced with increase in time and temperature. PhIP was not detected in control model systems where there was no heat applied. This indicates the requirement of heat to initiate the reaction for PhIP formation. Overall, temperature had a significant effect compared to time for PhIP formation throughout the system.

### 2.3. Effects of Amino Acid Precursors

Table 1 demonstrates the comparison between PhIP formation in proline and phenylalanine model systems detected at 12 min at all temperature. The results revealed the used of different amino acid precursors gave significant effects on PhIP formation. The amount of PhIP formed was significantly different between the proline and phenylalanine model systems. PhIP was significantly (*p* < 0.05) formed in the phenylalanine model system, which indicates phenylalanine as the major precursor for PhIP.

**Figure 3:**
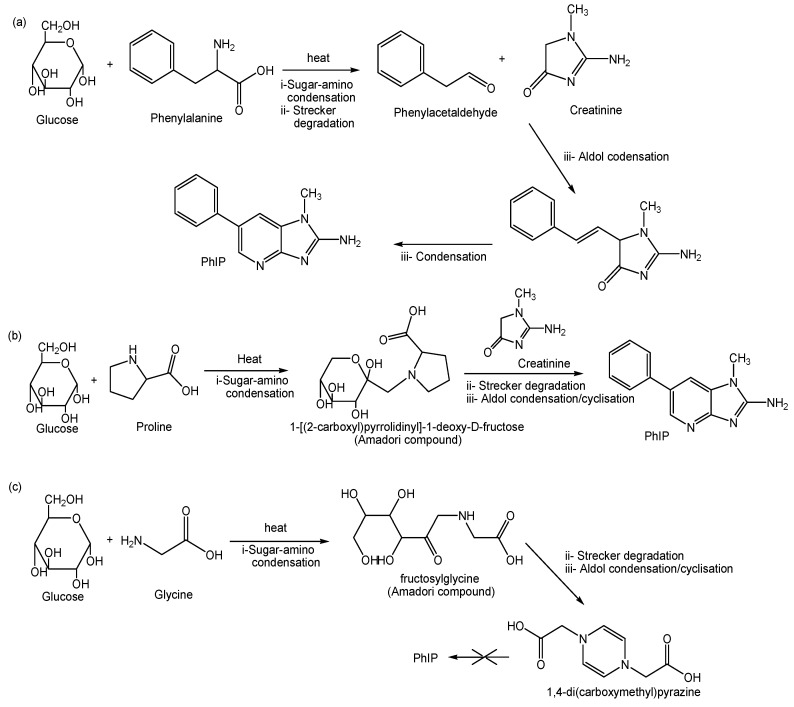
Formation of PhIP: (**a**) PhIP formation in the phenylalanine model system (modified) [30,31], (**b**) PhIP formation in the proline model system (modified) [32] and (**c**) PhIP formation in the glycine model system (modified) [33,34]. (i) Initial stage of the Maillard reaction, (ii) intermediate stage of the Maillard reaction, and (iii) final stage of the Maillard reaction.

All three amino acids have a unique chemical structure that might influence the formation of PhIP. The pathway formations for PhIP from different amino acids are shown in Figure 3. PhIP formed through the Maillard reaction involves three stages of reactions, namely the early stage (i), the intermediate stage (ii), and the final stage (iii) [28,29]. The early stage (i) was the sugar-amino condensation between the reducing sugar and the amino acids resulting in Amadori rearrangement products. In the intermediate stage (ii), the Amadori products undergo sugar breakdown and amino acids breakdown via Strecker degradation. The Strecker degradation steps involve the decarboxylation of amino acids in the presence of α-dicarbonyl, thus transforming the compounds into related aldehyde compounds, nitrogen containing compounds, small carbon fragments, and pyrazines [28,29]. In the final stage (iii) of the Maillard reaction, the intermediate from Strecker degradation undergoes an array of reactions including aldol condensation, dehydration, and cyclisation forming PhIP and other HCAs compounds [28].

Based on Figure 3, the aromatic structure of phenylalanine (C_6_H_5_CH_2_CH(NH_2_)COOH) that is formed by the benzyl side chain provides a pathway for forming phenylacetaldehyde (C_6_H_5_CH_2_C(O)H), an intermediate product that is required for PhIP formation [30]. The formation of phenylacetaldehyde occurred in the intermediate stage (ii) of the Maillard reaction via Strecker degradation. Throughout the Strecker degradation, phenylalanine undergoes decarboxylation and transforms into a structurally related aldehyde, which is phenylacetaldehyde [7,29]. In the presence of creatinine, phenylacetaldehyde undergoes an aldol reaction (iii) and forms an intermediate product. The subsequent condensation of the intermediate product leads to the formation of PhIP [7,30]. Other amino acids that share a similar molecular structure of R-CH(NH_2_)COOH with phenylalanine such as leucine (2(CH_3_)CHCH_2_CH(NH_2_)COOH), isoleucine (CH_3_CH_2_CH(CH_3_)CH(NH_2_)COOH), and tyrosine (OH(C_6_H_4_)CH_2_CH(NH_2_)COOH) were also shown previously to be able to form PhIP [25]. This may explain why PhIP was not formed in the glycine model system due to the short linear structure of glycine (NH_2_CH_2_COOH) [25]. Although there was no sign of PhIP formation, glycine was suspected to be the precursor for other HCAs compounds. Based on Figure 3, Kato et al. [33] identified 1,4-di(carboxymethyl)pyrazine cation radical from heating glycine with glucose. The compound was an important intermediate for formation of imidazoquinoxaline type-HCAs [33]. Formation of PhIP in the proline model system was in contrast with previous studies [21,35]. Nevertheless, previous studies have used different conditions for their model systems e.g., heating in water for 10 min at 180 °C, reflux in diethylene glycol at 128 °C for 2 h, and dry heating at 180 °C for 2 h. In this study, we showed that the cyclic structure of proline (NH(CH_2_)_3_CHCOOH) was able to form PhIP at higher temperature of 210 and 240 °C. Britt et al. [32] identified 1-[(2-carboxyl)pyrrolidinyl]-1-deoxy-d-fructose as the Amadori product from heating proline with glucose. Further cyclization or aldol condensation from the Amadori product causes PhIP formation.

Amino acids and reducing sugar are also the precursors for other HCAs and polycyclic aromatic hydrocarbons (PAHs). Besides PhIP, other compounds were also formed in proline and glycine model systems. This was the reason for the low amount of PhIP formed in the proline model system and no PhIP formation in the glycine model system.

### 2.4. Kinetic Formation of PhIP

Table 2 demonstrates the calculated constant for unknown intermediate (A), reaction rate constant (*k*), lag time (t_o_) and correlation coefficient (R^2^) using Equations (1) and (2). Based on the R^2^ values the good fit suggests the rate-limiting steps for the formation and degradation of PhIP in proline and phenylalanine model systems follows the first order model. As such, this result indicates that the reaction involving PhIP formation is influenced by the reactant molar concentration in which one of the reactants should be in excess [22]. Evidence has shown that many intermediates including imidazole and pyrazines were involved in HCA formation [7,17]. Therefore, many reaction steps are involved in the rate-limiting steps for the intermediates formation [22]. Both proline and phenylalanine model systems had t_o_ of 4.0951 min (180 °C) and 0.6403 min (150 °C), respectively. The t_o_ value continued t_o_ decrease rapidly to 0.00 min and 0.0334 min as the reaction temperature increased to 210 °C in proline and phenylalanine model systems. Ahn and Grün [23] also reported a decrease in PhIP concentration using a meat (beef) model system to study the kinetic formation of HCAs. The decrease of t_o_ value with increasing temperatures in proline and phenylalanine model systems indicates rapid reactions for PhIP formation at higher reaction temperature [24].

The rate constants (*k*) of PhIP formation were increased with increased heating temperature from 180 °C (0.0268 min^−1^) to 210 °C (1.1757 min^−1^) for the proline model system and 150 °C (0.0024 min^−1^) to 210 °C (0.0710 min^−1^) for the phenylalanine model system. The increase of *k* values showed fast PhIP formation in proline and phenylalanine model systems as mixtures were heated from 150 to 210 °C. At 210 °C, the proline model system showed rapid PhIP formation compared to the phenylalanine model system by having a *k* value of 1.175 min^−1^. The rapid formation was supported by the t_o_ value of 0.00 min. This result was in agreement with Moon and Shin [25] findings which reported an increase of *k* values with increased heating temperature in the phenylalanine model system. Ahn and Grün [23] reported a similar finding where *k* values were increased with increased temperature in a meat model system heated at a temperature ranging between 180 and 220 °C.

Degradation of PhIP was observed at high temperatures of 240 and 270 °C in both model systems. The rate of degradation (*k*) was determined by using the first-order model of *k* = −ln (A/A_o_)/t [27]. The degradation rate (*k*) increased from 0.0122 min^−1^ at 240 °C to 0.2897 min^−1^ at 270 °C in the proline model system and from 0.0258 min^−1^ at 240 °C to 0.0738 min^−1^ at 270 °C in the phenylalanine model system. Arvidsson et al. [22] discovered HCAs degradation using standard HCAs heated at 200 °C for 100 min and at 225 °C heated for 30 min. Chiu and Chen [27] reported PhIP degradation at 100, 150, and 200 °C by heating HCAs standard. The rate of degradation increased with increased heating temperature.

The temperature dependence on PhIP formation in each model system was predicted using Arrhenius, Equation (3) and Eyring equation, Equation (4). Table 3 demonstrates the calculated value of activation energy (Ea), activation enthalpy (∆H^‡^), and activation entropy (∆S^‡^) for each model system. Based on PhIP kinetic formation from Table 3, the proline model system had the highest Ea (114.12 kJ/mol) compared to the phenylalanine model system (95.36 kJ/mol). The low Ea in the phenylalanine model system indicates that the system requires a low energy level to initiate PhIP formation. Furthermore, the reaction was a non-temperature sensitive reaction as the reaction can proceed smoothly at low energy without increasing the temperature [25]. This was in contrast to the proline model system, which required high energy to initiate PhIP formation. The reaction was a temperature sensitive reaction where slight increase in temperature can influence the reaction as the molecules in the system had sufficient energy to form any reaction [22].

The change of energy from the reactant to the transition state was described by the Eyring equation, Equation (4). The ∆H^‡^ obtained for each model system was nearly equal to the Ea. This finding was in agreement with a previous study [23]. Proline and phenylalanine model systems reported ∆H^‡^ of 110.23 and 91.61 kJ/mol, respectively. Previous studies have reported ∆H^‡^ values for PhIP formation ranged from 72.4 to 134.4 kJ/mol using meat juice, meat, and chemical model systems [22,23,25,26].

Previous studies reported that the assumption on the mechanism can be estimated via ∆S^‡^ such as bimolecular (∆S^‡^ < 0) reaction or monomolecular (∆S^‡^ > 0) reaction [23,25,26]. Based on Table 2, both proline and phenylalanine model systems yielded negative ∆S^‡^ of −35.25 and −83.95 J/mol/K, respectively. The rate liming steps for the formation of PhIP through proline and phenylalanine as precursor was a pseudo-first-order (bimolecular), which involved two reactants with one reactant in an excessive amount [25]. The finding can be used to predict PhIP formation in foods as the result was in agreement with a previous study where a bimolecular reaction was identified in a meat juice model system (−213.08 J/mol/K) and phenylalanine model system (−145.49 J/mol/K) [23,25].

## 3. Materials and Methods

### 3.1. Chemicals

All solvents used in this study were of HPLC grade. Acetonitrile, methanol, phosphoric acid, and trimethylamine (99.5%) were purchased from Merck (Darmstadt, Germany). Glycerol, D(+)-glucose, L-proline (99%), glycine (99%), l-phenylalanine (99%), and creatinine were obtained from R&M (R&M Marketing, Essex, U.K). 2-amino-1-methyl-6-phenylimidazo[4,5-b]pyridine (PhIP) was obtained from Toronto Research Chemicals (Toronto, Canada). A stock solution of 100 ppm PhIP standard was prepared in methanol and kept at 4 °C.

### 3.2. Model Systems

The chemical model system as described by Skog and Jägerstad [36] was used with a number of modifications. Glycerol with 14% water was used as the solvent to maintain the reaction temperature at various points (150, 180, 210, 240, and 270 °C). The solvent was placed in a porcelain crucible and heated on a hot plate. Water was allowed to evaporate until the designated temperature was achieved. A thermocouple (Fluke, US) was used to monitor the temperature. A total of glucose (2 mmol), creatinine (4 mmol), and proline (4 mmol) were added into the crucible and mixture was heated for 4, 8, 12, and 16 min. The heated mixture (100 uL) was diluted in methanol and filtered through 0.22 μm nylon filter (Chromafil, Germany). The same experiment was repeated using phenylalanine and glycine precursors. The control sample was prepared by using the same set of aforementioned chemical model system without any heat treatment. All experiments were performed in duplicates.

### 3.3. Instrument

High-performance liquid chromatography, HPLC-FLD (Waters, Milford, USA) was equipped with fluorescence detector (Waters 2475, Milford, USA). The method was applied as described by a previous study [37]. The reversed phase TSK-gel column ODS 80-TM (5 µm particle size, 4.6 mm id × 250 mm) purchased from Tosoh Bioscience GmBH (Stuttgart, Germany) was used in this study. Detection of PhIP was performed at flow rate of 1.0 mL/min with 0.01 M trimethylamine at pH 3.2 (adjusted with phosphoric acid) as solvent A, 0.01 M trimethylamine at pH 3.6 (adjusted with phosphoric acid) as solvent B and acetonitrile as solvent C. The gradient program used was: 0–10 min, 5–15% C in A; 10–10.1 min, exchange A with B; 10.1–20 min, 15–25% C in B; 20–33 min, 25–55% C in B and at 33–55 min, column was rinsed with 55–5% C in A. PhIP peak was identified at ex307 and em370 using ambient temperature.

### 3.4. Reaction Kinetics

The kinetic formation of PhIP was described using different reaction equations. The formation of PhIP was described using the first-order reaction model [22,24]:

C = A(1 − e^−*k*(t − t^^o)^), (1)

where C is the concentration of PhIP (ng/mL), A is estimated maximum concentration of PhIP (ng/mL), *k* is the rate constant for the formation of PhIP (min^−1^), t is the heating time (min), and t_o_ is the lag time (min). Data were fitted into a non-linear regression model using Excel Solver to obtain the values of A, *k*, and t_o_. Degradation of PhIP at a specific temperature was determined using the following formula:

*k* = −ln(CA/CA_o_)/t, (2)

where CA is the concentration of PhIP after heating, CA_o_ is the initial concentration of PhIP and t is the heating time (min). The effect of temperature on PhIP formation was analyzed using the Arrhenius equation [23]:

*k* = Ae^−Ea/RT^, (3)

where *k* is the rate constant (min^−1^), A is pre-exponential factor, Ea is the activation energy (KJ/mol) for the formation of PhIP, R is the gas constant (8.3145 J/(mol/K)) and T is the temperature (Kelvin). The rate-limiting steps for the formation of PhIP in the different amino acids model system was determined by the activation entropy (∆S) using the Eyring equation [23]:

*k* = k_b_T/he^∆S^^‡/R^ e^−∆H^^‡/RT^, (4)

where *k* is the rate constant (min^−1^), k_b_ is the Boltzmann constant (1.381 × 10^−23^ J/K), h is the Plank constant (6.626 × 10^−34^ Js), R is the gas constant (8.3145 J/(mol K)), ∆S^‡^ is the activation entropy (J/mol K), ∆H^‡^ is the activation enthalpy (J/mol) and T is the temperature (K). Data were fitted into the Eyring equation by non-linear regression. The formation of PhIP was described by the calculated kinetic parameters including reaction rate constant, activation energy, entropy, and enthalpy.

### 3.5. Statistical Analysis

Minitab Version 16 (Minitab Inc. PA, USA) was used to analyze the data. A two-way analysis of variance (ANOVA) with Tukey test was used to evaluate the mean differences among the treatments applied for PhIP formation. The statistical significance was set at (*p* < 0.05) [38,39].

## 4. Conclusions

The formation of PhIP was significantly influenced by the reaction time, temperature, and types of amino acids. The increase in time and temperature significantly increased the PhIP concentration. However, at higher temperatures of 240 and 270 °C, PhIP formation was reduced in proline and phenylalanine model systems due to degradation. The results revealed that meat with high phenylalanine content had the highest tendency to form PhIP in high concentration. The formation of PhIP followed the first-order reaction suggesting the formation was influenced by the amino acids (reactant). Based on the kinetic parameters, the predicted pathway of PhIP formation was a bimolecular reaction, which involved a reaction between amino acids and sugar through the Maillard reaction. The results provide meaningful information on PhIP formation during heating. Several precaution steps can be taken by consumers to control PhIP formation during meat preparation or cooking which may help improve food quality and safety. Using a low temperature between 150 and 210 °C during cooking helps to reduce PhIP amount in cooked meat. Although PhIP amount reduces at high temperature (240–270 °C) due to degradation, using high temperature was not recommended as it may promote other HCAs and PAHs compounds. The kinetic result revealed PhIP formation was a bimolecular reaction, indicating that amino acids and sugar are required for PhIP formation. However, it is impossible to change the amino acids content in meat. Adding antioxidant ingredients during meat marinating such as exchanging sugar with honey may help to control PhP amount by neutralizing the intermediate and free radicals involved in PhIP formation.

## Figures and Tables

**Table 1:** PhIP formations in proline and phenylalanine model systems after 12 min of heating at 150, 180, 210, 240, and 270 °C.

Temperature, °C	Model System	
	Phenylalanine, µg/mL	Proline, µg/mL
150	0.28 ± 0.0004	ND
180	1.57 ± 0.04 ^a^	0.10 ± 0.0005 ^b^
210	12.08 ± 0.19 ^a^	0.36 ± 0.001 ^b^
240	19.50 ± 0.5 ^a^	0.41 ± 0.01 ^b^
270	7.47 ± 0.43	ND

^a,b^ Mean samples that share the same letter were significantly different (*p* < 0.05) between model systems. Mean ± standard deviation.

**Table 2:** The value of unknown intermediate (A), rate constant of formation (*k*), lag time (t_o_), and correlation coefficient (R^2^) from first-order model.

Model System	Temperature (°C)	A (ng/µL)	*k* (min^−1^)	t_o_ (min)	R^2^
Proline	150	nd	nd	nd	nd
	180 *	0.5098	0.0268	4.0951	0.99
	210 *	0.4371	1.1757	0.00	0.99
	240 **	-	0.0122	-	0.99
	270 **	-	0.2897	-	0.99
Phenylalanine	150 *	10.4238	0.0024	0.6403	0.98
	180 *	24.2223	0.0059	0.3715	0.99
	210 *	21.4828	0.0710	0.0334	0.99
	240 **	-	0.0258	-	0.97
	270 **	-	0.0738	-	0.95

* Values are calculated based on first order of C = A(1 − e*^−k^*^(t − t^^o)^); ** Values are calculated based on first order of *k* = −ln (A/Ao)/t; nd = not detected, there was no sign of PhIP formation based on HPLC analysis.

**Table 3:** The calculated value of activation energy (Ea), activation enthalpy (∆H^‡^), and activation entropy (∆S^‡^) of PhIP formation and degradation from proline and phenylalanine model systems.

Model System	Ea (kJ/mol)	∆H^‡^ (kJ/mol)	∆S^‡^ (J/mol/K)
Proline	114.12	110.23	−35.25
Phenylalanine	95.36	91.61	−83.95
